# Experiences and views of different key stakeholders on the feasibility of treating cancer-related fatigue

**DOI:** 10.1186/s12885-020-06858-6

**Published:** 2020-05-24

**Authors:** Claudia Canella, Michael Mikolasek, Matthias Rostock, Matthias Guckenberger, Josef Jenewein, Esther Linka, Claudia Six, Sarah Stoll, Roger Stupp, Claudia M. Witt

**Affiliations:** 1 Institute for Complementary and Integrative Medicine, University Hospital Zurich and University of Zurich, Sonneggstrasse 6, 8091 Zurich, Switzerland; 2 Hubertus Wald Tumorzentrum, University Cancer Center Hamburg, University Medical Center Hamburg-Eppendorf, Hamburg, Germany; 3 Department for Radiation Oncology, University Hospital Zurich and University of Zurich, Zurich, Switzerland; 4 Clinic Zugersee, Center for Psychiatry and Psychotherapy, Integrated Psychiatry, Triaplus AG, Oberwil, Zug, Switzerland; 5 Department of Oncology, University Hospital Zurich and University of Zurich, Zurich, Switzerland; 6 Zurich, Switzerland; 7 Cancer League Ostschweiz, St. Gallen, Switzerland; 8 Department of Neurology, Feinberg School of Medicine, Northwestern University, Chicago, IL USA; 9 Institute for Social Medicine, Epidemiology and Health Economics, Charité - Universitätsmedizin Berlin, Berlin, Germany; 10 Center for Integrative Medicine, University of Maryland School of Medicine, Baltimore, MD USA

**Keywords:** Cancer-related fatigue, Stakeholder engagement, Integrative treatment program, Complementary medicine, Qualitative study, Feasibility

## Abstract

**Background:**

Although cancer-related fatigue (CRF) has gained increased attention in the past decade, therapy remains a challenge. Treatment programs are more likely to be effective if the needs and interests of the persons involved are well represented. This can be achieved by stakeholder engagement.

In this paper, different key stakeholders’ experiences and views on the feasibility of treating CRF in the context of supportive care in hospital environments are analyzed.

**Method:**

In a qualitative study with the aim of developing an integrative treatment program for CRF, a total of 22 stakeholders (6 medical oncologists, 5 nurses, 9 patients, 1 patient family member, 1 representative of the Swiss Cancer League) were interviewed either in a face-to-face (*n* = 12) or focus group setting (*n* = 2). For data analyses, the method of qualitative content analysis was used.

**Results:**

The stakeholders referred to different contextual factors when talking about the feasibility of treating CRF in the context of supportive care in hospital environments. These included: assessment, reporting and information; treatability; attitude; infrastructure, time-management, costs and affordability; and integrative approach.

**Conclusions:**

Key factors of a feasible treatment approach to CRF are a coherent, cost effective integrative treatment program facilitated by an interdisciplinary team of health care providers. Furthermore, the treatment approach should be patient orientated, adopting an individualized approach. The major challenges of making the integrative treatment program feasible for CRF are resources and interprofessional collaboration.

## Background

Cancer-related fatigue (CRF) is a prevalent and complex syndrome in cancer patients and cancer survivors regardless of tumor and treatment type, and it is still “underreported, underdiagnosed and undertreated” [1]. A commonly used definition is provided by the National Comprehensive Cancer Network (NCCN): “CRF is a distressing, persistent, subjective sense of physical, emotional and/or cognitive tiredness or exhaustion related to cancer or cancer treatment that is not proportional to recent activity and interferes with usual functioning” [1].

There is broad agreement that CRF is difficult to treat with only a single intervention or medication prescribed alone, because of its multiple possible causative factors and the variable patterns of clinical expression in individual patients [2, 3]. Concerning the decision for suitable interventions, it is useful to keep in mind that approximately 51% of cancer patients use complementary medicine (CM) interventions and wish to include them in their further therapies [4, 5].

Therefore, a multimodal CRF treatment program combining pharmacological and non-pharmacological conventional and CM interventions seems highly promising.

How patients experience CRF has been adequately addressed in qualitative health research in the past years [6]. A review of Scott et al. discusses 154 published papers of qualitative studies between 1996 and 2009 that have described the patients’ experiences of CRF and the consequences for those affected by CRF or involved with its care. The studies showed relatively homogenous descriptions of the experience of specific sensations, of the impact of CRF on everyday life and of coping strategies, all of which were based on the levels of cognition, emotions, and physical expression and impact [6]. There was much less emphasis on researching the experiences of other stakeholders involved with CRF, such as caregivers and health professionals. In addition, participatory approaches such as stakeholder engagement were rarely applied to develop and assess treatment programs for CRF.

We developed an integrative treatment program for CRF using stakeholder engagement [7]. The treatment program has three different levels. The first level includes mandatory nonpharmacological interventions: information, motivation and exercise. The second level includes nonpharmacological choice-based interventions, such as mind-body medicine techniques, acupuncture and acupressure. The third level includes pharmacological interventions for severe CRF [7].

The treatment of CRF is first and foremost executed in the field of supportive cancer care [1]. In addition to the question of effective treatment options, there are different important contextual factors related to the feasibility of treating CRF, such as human and financial resources, infrastructure, institutionalization, geographical reachability, symptom management, prevention and views on the effectiveness of specific treatments.

In this paper, we present the results from the stakeholder engagement process asking about the contextual factors related to the feasibility of treating CRF in the experiences and views of the stakeholders, i.e., the people affected by CRF or involved in the treatment of CRF [8, 9] in supportive care in hospital environments. This study was conducted concurrently to the treatment program development study [7].

## Methods

In this section, we report the methods applied to answer the research question about the contextual factors related to the feasibility of treating CRF. For details about the approach for developing the integrative treatment program for CRF see Canella et al. 2017 [7].

### Stakeholder engagement

Key stakeholders were included to gather data about their experiences and views on the feasibility of treating CRF. They represented the following stakeholder groups: oncologists, radiation oncologists, psycho-oncologists, nurses, nurse experts (holding a Master’s degree or a PhD), representatives of a local Swiss Cancer League, patients, and patient’s family members.

To ensure that we integrated the key stakeholders in the study and that we obtained the relevant experiences with CRF as well as views on CRF [7], we recruited according to the principles of “theoretical sampling” [10]. This meant also to recruit participants from within and from outside the University Hospital Zurich.

From the group of the involved stakeholders, we formed a stakeholder advisory board. We aimed at involving one board member of every stakeholder group. We directly approached persons of whom we knew were very experienced with CRF to join the advisory board. We asked the different clinic directors of our oncology department as well as the nurse director and the representative of a local Swiss Cancer League. In addition, we asked one of our CRF patients to be part of the board. Due to availability issues, the patient family member unfortunately was not able to participate in the advisory board.

The seven board members all had experience with CRF and participated in the whole research process, which included face-to-face interviews, an advisory board meeting about the interpretation of the results and the further development of the integrative treatment program, and several written feedback rounds to reach consensus about the final treatment program.

### Qualitative data collection and analysis

The qualitative, participatory study was ethnographic in its nature [11–14]. Data collection and analysis followed the principles and methods of qualitative content analysis [11]. Qualitative content analysis involves identifying key topics, ideas and their relationships. In addition, formal characteristics of the data and their context are analyzed [11–14]. We applied qualitative content analysis to focus on the whole spectrum of topics and viewpoints that the stakeholders brought up regarding their experiences with CRF and the feasibility of treating it.

The stakeholders were interviewed either in a semi-structured face-to-face interview or in a focus group setting, both of approximately 90 min in duration [13–15]. They could choose to be interviewed at our institute, at their workplace or at their home. The interviewees were asked open questions about their experiences and needs concerning CRF and their opinions of the feasibility of treating CRF.

All interviews were audio recorded, transcribed verbatim and analyzed using qualitative data analysis software MAXQDA, version 11.1.2. We performed investigator triangulation to enhance the reliability of the analysis and the results [16]. In an iterative research process, two researchers analyzed the interview data independently and then discussed and revised their findings until a consensus was reached. The analyses consisted of an audio-club [17] where the researchers listened to the original audio-data in real time and then discussed their perceptions and interpretations with each other. Subsequently, a thematic coding in units of meaning was executed [10, 18] to identify and describe the topics, contextual factors and stakeholder experiences with CRF and its treatment [7].

## Results

### Participants

Overall, 22 stakeholders were interviewed (see Table 1).

**Table 1:** Stakeholder characteristics

Stakeholder groups	Total (***n*** = 22)	Gender	N	Mean Age (Range)
Patients	9	Female	7	55 (35–65)
		Male	2	
Patient’s family member	1	Male	1	33
Health care providers	12	Female	6	49 (34–64)
		Male	6	
oncologists			4	
radiation oncologist			1	
psycho-oncologist			1	
nurses/nurse experts			5	
representative of a local Swiss Cancer League			1	

With the health care providers, we conducted nine face-to-face interviews and one focus group consisting of one nurse and two nurse experts. On average, the health care providers estimated that approximately 57% (range 10%˗100%) of their cancer patients experience CRF.

With the patients, we conducted two face-to-face interviews and one focus group with seven participants.

The patients experienced different types of cancer and were in different stages of treatment after their diagnosis. They all combined standard cancer therapy and CM treatments. Between the patients’ cancer diagnoses and their CRF diagnoses, the average timespan was 10 months. In addition, a numeric rating scale with two questions was used to confirm the CRF diagnosis of the participating patients at the beginning of the project [7, 19]. For further details about the patients cancer characteristics see Canella et al. [7].

The stakeholder advisory board was formed out of the 22 stakeholders. The seven individual board members were interviewed face-to-face as stakeholders independent of the advisory board meetings [7].

### Contextual factors related to the feasibility of treating CRF in supportive care in hospital environments

The stakeholders referred to different main topics when talking about the feasibility of treating CRF: assessment, reporting and information; treatability; attitude; infrastructure, time-management, costs and affordability; and integrative approach.

Selected original quotes representative of the addressed topics and views during the interviews are presented in Table 2. In addition, all the terms in quotation marks indicate original quotes from the stakeholders.

**Table 2:** Selected stakeholders’ original quotes representative of the addressed topics and views during the interviews

	Stakeholders original quotes
*Assessment and Reporting*	Radiation oncologist: “From my view, we do not ask/ nor use a systematic assessment [of CRF] at the moment, no quantification”.
	Oncologist: “Everyone has their own technique what is been asked and how you ask. And then, it is difficult asking things only to record it but not having a solution for it”.
	Psycho-oncologist: “The problem with CRF is that it does not appear at a certain point in time … it can happen at any point during the course [of cancer disease]. And we do not know 100%, as far as I know, what the causes are … and which factors play a role in it.”
	Psycho-oncologist: “The big challenge is to distinguish [CRF] from other psychiatric diseases, above all from depression, but also from anxiety. That is a big diagnostic and therapeutic challenge”.
	Nurse expert: “You could delegate it to the nurses because the medical doctors always have so little time. I mean the assessment [of CRF] … if you had an algorithm of what data, clinical parameters and so on to gather … that could be an interesting job for an advanced nursing practitioner”.
	Radiation-oncologist: “That [addressing fatigue symptoms] is not in the foreground in the patients. Surviving is in the foreground in the patients who are really in the middle of a cancer therapy. Then, the severe side effects, acute side effects that come from radiation. So, fatigue does not occupy a big space neither in the informative consultation nor in the consciousness of the patient”.
*Treatability*	Oncologist: “It is a bit frustrating. You try to help, trying to improve some things, but mostly, time must just pass by and it gets a little better in the end. We don’t have good therapy options available.”
	Radiation-oncologist: “We do not really have a specific systematic therapy of CRF within our clinic treatment guidelines … .nor do we have a specific offer [for the patients].”
	Nurse expert: “Concerning therapy strategies, I see a bit a split up between physical, emotional, mental and I would also add spiritual and of course complementary methods … ”
	Nurse expert: “People are telling me that they cannot trust their own body anymore because they suffer from cancer and did not notice it. Afterwards, the fatigue and exhaustion kick in because of the therapy. And then there are the surgeries, being disfigured, not being able to find yourself beautiful anymore. Feeling a distance to their own body, looking from the outside to the own body and saying, this is someone else. I don’t want anything to do with this …. And then you should return to life and it does not work … You cannot get out of your own way forever … if you want to go for a swim or the hair is grey all of a sudden. It is really important that the people get back these abilities, that they are able to influence their acts and experiences … ”
	Patient family member: “… it is searching for a dialogue and rediscovering the awareness of the own body … sensing yourself … and trying to create a sense of achievement together … ”
	Patient: “How do you fight fatigue?...If you are in it, you cannot make it. The exhaustion makes it impossible to move … and then, there is the pain … finally, you resign, and then the spiral spins down fast … when I am inside the fatigue or in this vicious circle, I do not think that I am capable of anything … It would be important to begin early in the therapy with physiotherapy to avoid physical imbalances and physical decline … ”
*Attitude*	Patient family member: “Something that you can do by yourself. Nothing that is additionally inflicted upon you or is being done to you from the outside … Something that I can contribute to the whole. I believe that this should lead to a certain kind of self-confidence, that you are able to do something and that you can do something good to yourself in this time.”
	Oncologist: “Empowerment. Keep them in the driver seat.”
	Nurse expert: “Individualizing and prioritizing … for [treating] very exhausted people … when you do individualize, you automatically determine priorities … ”
	Patient: “After the disease, I would have wished that the hospital told me what I can do against the fatigue … I googled a bit, but in this situation, you are still so tired and everything needs so much energy. Life alone costs so much energy … you are more reserved, and you are not in the mood to try things. I would have wished to receive some addresses or similar things … something where I would have been accompanied … how do I cope with … the whole fatigue … and what can I do against it?...You are so tired and without energy that you are happy when others decide for you … because it has to do with effort … I would have wished different options and offers open to choose from what I wanted … ”
	Patient: “After the active cancer treatment … you are discharged, then you have to fend for yourself … you are pretty much left alone”.
	Oncologist: “Certain cancer patients that have never learned to care for themselves … you cannot expect them to jump on and say: Yay, now I do something for myself! … They don’t turn around 180 degrees and act completely different than their 50, 60 years before … ”
*Infrastructure, time-management, costs and affordability*	Oncologist: “If we want it to be done [establishing a CRF treatment program], we need a point of care. That means, on the one hand, the medical doctor and, on the other hand, the nurses who are near the patients … It means you need rooms within the clinic, as near as possible, as visible as possible … in an ideal situation … We are way too disparate … people are too far away … The patients have to gather together different offers. That is not always easy. It is like in the supermarket where they put the chocolate things before the cash desk. You consume of what you know to exist. You seduce by being there.”
	Oncologist: “The information has to be done by the point of care … I say by the nurses first … because they have a longer exposure to the patients … there are other points of contacts where topics can be addressed that usually fall short...The information also has to be there, ideally at a desk where you can get the information or patients can ask about while they are waiting … or while passing by when leaving [the hospital] … If I could build a hospital, I would want a shopping center … with psycho-oncology, social services … cancer league … a welcome desk with brochures and information material, a wigmaker … and so on and so on … ”
	Nurse expert: “They [cancer patients] often have some physiotherapy … or a psycho-oncological consultation or a follow up or the baby-sitting that didn’t work and then another appointment follows and another and another and another. Or they have long ways … It is not to be underestimated, because the survivors are tired. And they have cognitive dysfunctions and they are exhausted afterwards and know, when the concentration [of an appointment or intervention] is behind them, they have consumed up all their energy for the whole day”.
	Radiation-oncologist: “After the motto, more is better, I do not believe in this. I do not consider it as useful applying five different methods to attack CRF...One method for sure. Two, ok. I would not expect the patients to do more.”
	Patient: “Maybe the psycho-education would have been feasible if you say, ok, today after or before the chemo you have another hour. How much outcome you would have in doing it that way, I don’t know. How receptive you would be, also cognitively. This is another thing that you are not receptive at all. Maybe it [the treatment program] is feasible if it is integrated into the proceedings of the hospital … ”
	Nurse expert: “Maybe we could create an offer for people who do not live on the sunny side of life. Some foundation or donation accounts … to support something.” … “I would connect it to the indication...In case of this diagnoses maybe one part would be funded. That would be useful … and then probably more evidence is needed.”
	Oncologist: “There is this consumerism. I think, it would be good if the people must pay a bit more because that causes another identification.”
*Integrative approach*	Nurse expert: “They [the patients] really came and asked, what can we do? Additionally, complementary? What offers are there? What would help me?”
	Psycho-oncologist: “Personally, I really like complementary medicine, because it … offers something for the patients that helps them. Personally, I prefer that patients who suffer from psychological problems, who are stressed, that they learn something active, how they can create their life themselves again … And patients love complementary medicine anyway because they feel that it is something good for them and that it helps them and does not harm.”
	Nurse-expert: “We have some [nurses] who are good in the complementary medicine approaches, who are vocationally educated in it. We also refer [patients] to your clinic [Institute for complementary and integrative medicine]. We often do this. I think it is strongly growing … there is a tendency. Now, it is more in the heads of the medical doctors and the nurses.”
	Patient: “We, the patients, have to initiate and build it. There is no net of connections or networking among the medical doctors yet. And this is something where both sides could benefit from one another, and in the end, the patient has a huge benefit from it. I really cannot understand why they don’t do it.”
	Nurse expert: “What I think is that it is mostly a single element [from a complementary medicine approach]. Therefore, it is not a package where you could choose something and that is harmonized to one another. It is rather that the patients try something because he has heard of it or someone recommended it. He just tries and either it is good or not. It is complex. I think, most [patients] try something.”
	Nurse expert: “We consider ourselves as scientists, natural scientists … I tell [my patients] that my belief system differs from these [complementary medicine approaches], but that I am – of course – full of respect and acceptance for these methods as long they don’t harm themselves.”

### Assessment, reporting and information

The medical doctors, nurses and nurse experts stressed the challenge of assessing CRF and asking their patients about CRF-related symptoms periodically. The health care providers usually did not use a standard diagnostic tool for assessing CRF. “Everyone had more or less an individual approach” (medical oncologist) to ask their patients about symptoms of CRF. It was a challenge for the health care providers to keep track of CRF in their patients because CRF can have multiple causes, can appear in very different stages of cancer and can differ in its clinical expression in the individual patients. The psycho-oncologist added that it is often difficult to differentiate between CRF and other psychological conditions, such as depression or anxiety. A nurse expert linked the topic of assessment and reporting with the observation that medical doctors are often too overburdened with the standard cancer therapy consultation to execute a systematic CRF assessment. She suggested that a systematic implementation of a standard diagnostic tool for CRF might be more feasible if an advanced nursing practitioner could take over that task from the medical doctors.

Some health care providers reported that their patients rarely addressed problems or symptoms of fatigue during the consultation because they were focused on surviving and on the more acute side effects of active cancer therapies.

Patients felt that they were not informed specifically enough about how to treat CRF. They felt like they had to search for treatment options on their own what was challenging while experiencing simultaneously CRF. They wished they could have been monitored for CRF throughout the active cancer therapy but especially after active treatment. The advisory board discussed when would be a good point in time to inform the patients about CRF. They agreed that cancer patients should be informed early after their diagnoses and reflected on the possibility of creating an online information tool for the patients.

### Treatability

The health care providers often did not see good results when trying to treat CRF. They also mentioned that there was no specific treatment for CRF in their hospital at the time of the interview. It was a problem for the health care providers that CRF could not be treated with a single, simple and effective intervention, so they opted for interdisciplinary collaboration and an integrative treatment program when approaching the treatment of CRF. A nurse expert expressed the need for approaches at different levels, including the physical, emotional, mental and spiritual levels.

Many stakeholders also reflected on the goal of CRF treatment, which they saw as increasing the energy level of the patients, improving their quality of life and helping them adopt coping strategies. A nurse expert differentiated between patients in curative and palliative situations. To her, the goal in palliative patients should be “coping” (nurse expert) and “managing their own energy levels” (nurse expert) throughout the day, whereas in a curative setting, the patients should aim to regain their energy mainly by exercising. Most stakeholders stressed that one of the most important goals is to provide patients with options that help them regain trust in their own bodies and in their abilities, as many cancer patients lose faith in their own bodies. For the stakeholders, this was also strongly linked with the topic of regaining self-efficacy. The stakeholders thought that this could be best achieved by exercising and by psychoeducation. However, it is a major challenge for people with CRF to become active on a regular basis because being active and experiencing CRF are contradictory in their nature. The stakeholders differed in their views on how to approach this challenge. Whereas some patients would have preferred personal coaching with an individual workout program, a few health care providers opted for group trainings tailored for CRF patients, with the focus not only on the financial and infrastructural feasibility but also on the possible benefits of the group setting, such as sharing similar diagnoses, exchanging experiences and motivating each other.

### Attitude

The stakeholders agreed on the attitudes of the patients and health care providers needed for a successful integrative CRF treatment program. The treatment approach should be patient-oriented, should focus on self-care options and should create possibilities for self-management for the patients. According to the stakeholders, a patient-oriented approach is also needed to strengthen patients’ self-efficacy and to overcome feelings of helplessness that often go along with experiencing a life-threatening disease such as cancer.

The nurses and nurse experts stressed that an individualized approach is needed. This would consider patients’ cultural and social backgrounds as well as their individual experiences with their cancer and with CRF, recognizing the importance of connecting with the patients’ resources and interests.

The patients unanimously agreed with the patient orientation, but also wished to be simultaneously informed, monitored and accompanied by health care providers because they felt unable to act completely autonomously while experiencing deep exhaustion and fatigue. They often felt they were being left alone with their CRF, especially after active cancer treatment.

Some health care providers pointed out that an integrative approach and a focus on self-care often require a change in health behavior in the patient and that this is a serious challenge while experiencing cancer and CRF.

### Infrastructure, time-management, costs and affordability

The health care providers identified a need for a multimodal approach to treat CRF and talked about the consequences that come along with such an approach. First, hospitals are always short of manpower, infrastructure and time to meet all the different needs of individual patients. In addition, it is a challenge to coordinate the treatment between different departments and ensure the flow of information between all involved parties. Some stakeholders imagined a “shopping-center” (medical oncologist) or “drop-in-center” (medical oncologist) where the different treatments would be coordinated, monitored and located in the same building.

Normally, cancer patients have many appointments that can result in an overload of consultations and therapies. Consequently, most stakeholders opted for a prioritization of treatments – also based on the severity of CRF in the individual patient – and a focus on options that could be executed at home, such as exercising or acupressure. According to the stakeholders, prioritizing is even more important for CRF patients because they experienced these patients to be very restricted on all levels. Too many appointments limit the processing of information and interventions in CRF patients. Therefore, a good organization of the appointments is needed as well as locating therapies within a comfortable geographical distance from where the patients live. In addition, some stakeholders pointed to the challenge of coordinating work with an extensive treatment program, as is often the case in cancer patients.

The stakeholders agreed that the integrative treatment program would be most feasible if it would be fully covered by public health insurance. Admission into public health insurance usually requires standard diagnostic tools and evidence from good quality randomized controlled clinical trials for the interventions; both would be currently available. However, there were also some opinions from health care providers that patients should pay privately to increase their adherence to the treatment program. Simultaneously, they opted for establishing a “social welfare fund for cases of hardship” (medical oncologist). The nurse experts pointed out that cancer survivors usually struggle financially because they have lost their jobs or cannot work anymore because of their cancer.

### Integrative approach

All patients wished to have an integrative approach to their therapies. They asked their medical doctors or nurses about what complementary medicine (CM) interventions they could add to their therapy.

In general, the health care providers considered CM approaches as especially supportive for their patients, contributing actively to their recoveries. They noticed a growing awareness in medical doctors and hospital environments of the possibility of adopting an integrative approach and referring patients to the respective institutions. However, patients complained about a lack of interdisciplinary collaboration between the different health care providers.

The health care providers did not have the impression that their patients followed a coordinated integrative treatment program at the time of the interviews. Instead, they experienced isolated applications of single CM interventions in their patients, such as yoga, diets or phytomedicine, which had been recommended to the patients by their private environments or were found on the internet.

The health care providers themselves only recommended CM interventions for which they personally had a clear idea of the benefits. Some of them adopted a stance of not believing in certain interventions but thinking “even if it is not effective, it does not harm” (nurse expert). Some health care providers were critical about the effectiveness of CM interventions and opted for strict “academic, scientific, evidence-based complementary medicine” (radiation-oncologist).

First experiences with the implementation of the integrative CRF treatment program in our clinic point to the program being feasible if patients come into the clinic with CRF as their main complaint. However, more often, CRF is one of many complaints that cancer patients report when coming to our clinic. Our medical doctors then prioritize the interventions with the patients. Exercising and mind-body medicine techniques are discussed with every CRF patient. Then, usually one other intervention from the program for CRF is selected, often acupuncture or acupressure. In addition, procedures or remedies which cover both CRF and the patients’ other symptoms are selected, such as mistletoe extract or other herbal drugs.

## Discussion

Table 3 provides a summary of barriers and facilitators regarding the feasibility of treating CRF in supportive care in hospital environments in the views of the stakeholders.

**Table 3:** Summary of barriers and facilitators regarding the feasibility of treating CRF in supportive care in hospital environments in the views of the stakeholders

	Barriers	Facilitators
*Assessment*	⋅ No standard diagnostic tool	⋅ Standard diagnostic tool
	⋅ No systematic CRF assessment	⋅ Systematic CRF assessment
		⋅ Patient orientation
*Reporting*	⋅ CRF has multiple causes	⋅ Encouraging patients to report CRF symptoms
	⋅ CRF differs in its clinical expression in the individual patients	
	⋅ Patients rarely address symptoms of fatigue on their own accord	
*Information*	⋅ No specific systematic information of patients about CRF	⋅ Information about CRF early after diagnoses
		⋅ Online information tool
*Treatability*	⋅ Not treatable with a single intervention	⋅ Interdisciplinary collaboration
	⋅ No treatment guidelines in the hospital	⋅ Integrative treatment program
		⋅ Monitoring
		⋅ Working towards self-efficacy of the patients
*Attitude*	⋅ Feelings of helplessness	⋅ Patient-orientation
	⋅ Experiencing a life-threatening disease	⋅ Individualized approach
		⋅ Self-Care options
		⋅ Self-management
*Infrastructure*	⋅ Hospitals are short of infrastructure	⋅ Drop-in-center (coordination, monitoring)
*Time-management*	⋅ Doctors and patients are short of time	⋅ Prioritization of treatments
	⋅ Overload of consultations and therapies	⋅ Focus on self-care
	⋅ Coordination of work and treatment	⋅ Geographically reachable treatment options
*Costs and affordability*	⋅ Hospitals are short of manpower	⋅ Coverage by public health insurance
	⋅ No coverage by public health insurance	⋅ Social welfare funds
*Integrative approach*	⋅ Lack of interdisciplinary collaboration	⋅ Evidence-based integrative medicine approach
	⋅ Lack of coordinated integrative programs	⋅ Integrative treatment program

Although all the discussed topics are linked to different challenges of making the suggested integrative treatment program feasible for CRF, we identified two major challenges out of the interviews with the stakeholders: first, staff and financial resources, and second, the coordination of such a multimodal, interprofessional approach in terms of infrastructure, flow of information and manageable organization. To date, supportive cancer therapies are still heavily competing with antineoplastic therapies for limited health care funding [20]. As the financial burden of cancer care increases in the future [21–23], health care funding remains a major challenge for supportive cancer therapies. Increasing reimbursement for supportive therapies would require a considerable shift in the rating of the importance of supportive care compared to antineoplastic therapies by governments, health care policy makers and investors [20, 24]. However, as cancer survivors are a rapidly growing population, awareness of the need for supportive cancer therapies increases simultaneously. Not only the financial burden of cancer disease in patients has to be considered, but also the cognitive, physical, emotional and spiritual burdens and their impacts on productivity, citizenship and contribution to society [20, 25]. Apparently, there is no global solution either for solving infrastructural problems or for the coordination of a multimodal treatment approach and interprofessional collaboration. There is a broad consensus that interprofessional collaboration in supportive cancer care ideally must entail a united team effort of “medical, nursing, psycho-social and spiritual support into a global and anticipated team approach to the patients and their social environment” ([26], p.10). However, practical solutions must be found and implemented locally, ensuring the transfer of knowledge and professional expertise [25, 27]. To overcome the gap between research and implementation into practice, key stakeholders – including scientists, health care policy makers, funders, health professionals, advocacy groups, patients and caregivers – have to collaborate on the basis of “trust, mutual respect and shared responsibility” ([25] , p.580).

### Potential approaches

In our clinic, we address these challenges in various ways. As we are part of the University Hospital Zurich all our supportive cancer care interventions are covered by the public health insurance. This enables almost all patients to have access to our treatment options. We handle the challenge of the coordination of such a multimodal, interprofessional approach in different ways. We built an interdisciplinary team of medical doctors with different integrative medicine expertise, psychotherapists, nurses and a nutritionist in our clinic. The whole clinical team regularly meets for case conferences where the treatment of single patients is discussed and coordinated.

A promising attempt to approach the challenge of a globally standardized treatment of CRF is the implementation of evidence-based guidelines for CRF management, such as the NCCN guideline [1] or the CAPO guideline [28]. Assessments of the guidelines showed that they face similar problems concerning their feasibility: availability of the guidelines and diagnostic tools, education of health professionals and patients, coordination of an interprofessional team, integration with existing practices and patient-orientation (e.g. [29]).

### Strengths and limitations

The study has strengths and limitations. We gathered the experiences and views of CRF patients and included other key stakeholders, such as health care providers or patient family members, to obtain a broader perspective towards an integrative treatment approach to CRF, especially considering the contextual factors that influence the feasibility of treating CRF. In addition, we adopted a participatory approach by working with a stakeholder advisory board. A limitation of the study is that not all stakeholders were equally included, and some were even missing completely, such as allied health practitioners, hospital administrators or health insurance representatives. Other stakeholders might have provided a broader perspective. Another limitation is that we conducted the study in a hospital environment that might not be applicable to other health care environments.

Future research should include the scientific evaluation of the integrative treatment program for CRF, addressing especially the relevant contextual factors, infrastructure, resources, cost-effectiveness and interprofessional collaboration.

## Conclusion

Key factors of a feasible treatment approach to CRF are a coherent, cost effective integrative treatment program facilitated by an interdisciplinary team of health care providers. Ideally, such a program should entail a coordinated monitoring of the treatment of the patients. Furthermore, the treatment approach should be patient orientated. Adopting an individualized approach, health care providers should aim at self-efficacy of the patients.

Despite the numerous barriers, a clinic of integrative medicine embedded in a hospital environment and with a focus on supportive cancer care has the potential of realizing such a feasible integrative treatment program for CRF.

## Data Availability

The datasets generated and/or analysed during the current study are not publicly available due research participant privacy, but are available from the corresponding author on reasonable request.

## Abbreviations

CM: Complementary medicine
CRF: Cancer-related fatigue
NCCN: National Comprehensive Cancer Network
